# Examining the impact of a health report card on follow through with fall risk recommendations: an observational study

**DOI:** 10.1186/s12877-024-04686-y

**Published:** 2024-02-16

**Authors:** Abigail L. Kehrer-Dunlap, Rebecca M. Bollinger, Brianna Holden, Beau M. Ances, Susan Stark

**Affiliations:** 1 Program in Occupational Therapy, Washington University School of Medicine, 4444 Forest Park Ave, 63110 St Louis, MO Box 8505, USA; 2 Department of Neurology, Washington University School of Medicine, St. Louis, MO USA

**Keywords:** Older adults, Falls, Occupational therapy, Health report card

## Abstract

**Background:**

Increasing older adults’ awareness of their personal fall risk factors may increase their engagement in fall prevention. The purpose of this study was to explore the impact of and participant satisfaction with a comprehensive occupational therapy fall risk screening and recommendations for evidence-based fall prevention strategies based on personalized fall risk results for community-dwelling older adults.

**Methods:**

Cognitively normal participants (Clinical Dementia Rating = 0) were recruited from an ongoing longitudinal study of memory and aging. Participants completed 2 annual in-home visits, fall risk questionnaires, and 12 months of fall monitoring between visits. Participants received a health report card with their fall risks and tailored recommendations in 6 domains. Participants completed follow-up questions at their next annual in-home visit about the fall risk recommendations and their satisfaction with receiving their fall risk results.

**Results:**

Two hundred five participants completed 2 annual visits and 12 months of fall monitoring. Of the 6 domains of recommendations provided, participants were most likely to follow through with getting an annual eye exam and reviewing their medications with their doctor or pharmacist. Older adults who fell were significantly more likely to receive recommendations for finding fall prevention classes (*p* = 0.01) and having a doctor or pharmacist review their medications (*p* = 0.004). The majority of participants were satisfied receiving their fall risk results (92%) and believed it to be beneficial (90%), though few participants shared their results with their doctor (20%).

**Conclusions:**

An occupational therapy fall risk screening and tailored recommendations were not sufficient to encourage follow through with fall risk recommendations. Older adults may benefit from additional support and encouragement to reduce their fall risk. Additional research is needed to examine awareness of fall risks and follow through with fall risk recommendations among community-dwelling older adults.

**Supplementary Information:**

The online version contains supplementary material available at 10.1186/s12877-024-04686-y.

## Background

Nearly 30% of older adults in the United States experience a fall each year [1]. Falls are the leading cause of injury among older adults, including sprains, fractures, and head injuries [2]. Regardless of injury, older adults who have fallen may also experience psychological or emotional consequences, such as fear of falling and decreased self-efficacy [3]. Fall risk factors are multifactorial, and treatment should be tailored to individual risk profiles. To address the detrimental effect of falls on an increasingly older population, fall prevention programs and strategies have been developed to promote safety and allow older adults to remain in their homes [4, 5]. Evidence-based approaches to address fall risk factors may include exercise programs to improve balance and strength [4, 5], removing hazards in the home [5, 6], and reducing the number of prescription medications [4]. Home assessments provided by an occupational therapist, including the evidence-based strategies listed above, can reduce the risk of falling and may encourage older adults to make changes to promote safe aging in place [7].

Despite the importance of reducing one’s risk of falling, older adults may not participate in fall prevention programs and studies for several reasons. Some older adults may believe that falls are a consequence of normal aging, that they are not personally at risk, or that reporting a fall may imply functional decline [8, 9]. For example, less than half of older adults report discussing previous falls with their primary care physician [10, 11]. Additionally, some older adults at risk for falling may overestimate their abilities or may not believe that they would benefit from a fall prevention program [12]. Other barriers may include older adults’ lack of time, access, or finances to engage in fall prevention programs and studies [13, 14]. Thus, efforts are needed to increase older adults’ awareness of fall risks and the importance of fall prevention even if they have not experienced a fall yet.

One strategy to increase older adults’ participation in fall prevention is to provide them with a fall risk screening and their personalized results [15]. This has the potential to increase older adults’ awareness of their own risk, encourage engagement in fall prevention behaviors, and allows them to share results with health care providers to initiate discussions about fall risks and possible approaches for preventing falls [15]. Therefore, we developed a health report card (HRC) of evidence-based fall risks in 6 domains to inform participants of their personal fall risks and tailored recommendations that may reduce their risk of falling [16]. The HRC may encourage older adults to make changes to prevent future falls and has potential to increase participant satisfaction. The purpose of this study was to explore the impact of and participant satisfaction with a comprehensive occupational therapy (OT) fall risk screening and recommendations for evidence-based fall prevention strategies for community-dwelling older adults.

## Methods

### Participants and study design

This analysis utilized data from an ongoing longitudinal cohort study with community-dwelling older adults from the Knight Alzheimer Disease Research Center (ADRC) at Washington University in St. Louis, further details of which have been published previously [16]. Briefly, participants who met inclusion criteria were approached by Knight ADRC staff at the time of their annual clinical assessment. Interested individuals were referred to a study team member who provided information on the longitudinal study and obtained written informed consent in the home prior to collection of study data. The purpose of this longitudinal study was to identify whether functional mobility and falls could serve as preclinical markers for Alzheimer disease [16]. Participants included in the longitudinal cohort study were: (1) ≥ 65 years old and (2) cognitively normal (Clinical Dementia Rating [CDR] [17] score of 0) at their most recent clinical visit at the Knight ADRC. Participants completed annual in-home screenings of fall risk factors with an OT practitioner and monthly fall monitoring for 4 years and received the fall risk screening and recommendations as part of a research participation incentive.

This retrospective analysis includes all participants enrolled in the longitudinal study who completed 2 annual in-home screenings and 12 months of fall monitoring between these home visits. This study was approved by the Institutional Review Board at Washington University in St. Louis (reference number: 201807135).

### Annual home visit and fall risk screening

Participants received annual in-home screenings of fall risk factors, including balance and gait, functional mobility, sensation, and environmental hazards. Participants completed annual questionnaires of additional fall risk factors, such as fear of falling, via electronic survey or telephone interview. After the annual questionnaires and in-home visit were completed, participants received an HRC by mail that included information about their fall risks, explanation of scoring for measures used to evaluate fall risks, and tailored recommendations to reduce their risk of falling. Participants with one or more fall risks identified in the OT fall risk screening were encouraged to share their HRC with their primary care physician to initiate conversations about their personal fall risks and possible preventative measures.

During the next annual in-home visit, participants reported whether they had followed up on recommendations in each applicable domain over the past year. They also rated their satisfaction with receiving their fall risk results, the degree to which they found this information beneficial, and whether they had shared their fall risk results with their primary care physician.

Due to the COVID-19 pandemic, all in-person research activity was paused for nearly 15 months. Participants who did not have an in-home visit during their second year in the study completed follow-up questions about their Year 1 recommendations during their Year 3 home visit. Participants who enrolled during the COVID-19 pandemic completed follow-up questions during their Year 2 home visit.

### Fall risk measures and recommendations

Participants received HRCs that included their fall risk results based on the OT screening and tailored recommendations for reducing their risk of falling. Tailored recommendations and the rationale for the 6 domains are as follows: (1) impairments in *balance and lower extremity strength* have been associated with gait deviations and increased risk of falling [5, 18]; (2) low *vision* and impaired contrast sensitivity increase the risk of experiencing one or multiple falls [18]; (3) *fear of falling* is associated with increased risk of falling and limited activity participation [3, 18]; (4) removal of *home hazards* reduces the rate of falls in older adults [6]; (5) impaired lower extremity *sensation* increases one’s risk of falling [19]; and (6) polypharmacy and taking 4 or more prescription *medications* can increase one’s risk of experiencing one or multiple falls [3, 20]. Follow through with recommendations was measured by the number of recommendations the participant followed out of the total number of recommendations provided. Table 1 displays a summary of fall risk domains, measures, established cutoff scores [3], and recommendations. Participants whose scores fell below cutoff values for each fall risk domain were classified as not having a fall risk and did not receive tailored recommendations.

**Table 1:** Recommendations and clinical cutoff scores for fall risk domains

Fall risk domain	Measure	Clinical cutoff scores [3]	Recommendation provided
Balance and strength	Tinetti Performance Oriented Mobility Assessment (POMA) [25]	< 25/28	“If you feel unsteady when standing or walking, consider exercises that improve balance and strength, like Tai Chi.”
	30-second Chair Stand Test (CST) [26]	Fewer stands than norm-referenced scores for age group	
Vision	Near contrast visual acuity using the King-Devick Apple iPad App [27]	≤ 20/40 or worse	“If you experience changes in your vision, get an annual eye exam and replace your glasses as needed.”
	Low contrast visual acuity using the King-Devick Apple iPad App [27]	≤ 20/40 or worse	
Fear of falling	Short Falls Efficacy Scale-International (FES-I) [28]	> 10	“If you are worried about falling, talk to your doctor about fall prevention classes, and tell them right away if you fall.”
Home hazards	Westmead Home Safety Assessment [29]	≥ 4 hazards	“If there are fall hazards in your home, use the home safety self-assessment tool to find and fix fall hazards to make your home safer.”
Sensation	Physical assessments (vibration and sharp sensation) [30]	Vibration: <10 s	“If you experience tingling, numbness, or pain in your feet, ask your doctor to check your feet at least once a year.”
		Sharp: any impairment	
Medications	Number of prescription medications	≥ 4 prescription medications	“If you take more than 4 prescription medications, have your doctor or pharmacist review your medications, including over-the-counter medications and vitamins.”

### Fall monitoring

Falls were defined as an unexpected event in which the individual came to rest on the ground, floor, or a lower level [19]. Participants were encouraged to record falls using a daily calendar-journal [21]. Falls were reported to study staff via automated phone call or e-mail survey monthly [21]. Participants received an incentive via gift card for each month of fall reporting [21]. If a participant reported a fall, a trained rater followed up via phone to collect additional details about the fall. Falls included in this analysis were reported for the 12 months following the participant’s Year 1 home visit.

### Statistical analysis

Type of recommendation and follow through were compared for individuals who fell versus those who did not using Chi-square tests (see Supplementary Table 1). Significance level was set at 0.05 for between-group comparisons of participants who fell versus those who did not fall. A frequency analysis was used to examine satisfaction with receiving the HRC and circumstances of reported falls. Data were analyzed using R v. 4.2.1 [22].

## Results

Two hundred five participants completed 2 in-home visits and 12 months of fall monitoring as part of the ongoing study and were included in this analysis. Participants reported follow through with Year 1 recommendations at Year 2 (*n* = 57) and Year 3 (*n* = 148). Participants were, on average, 74.8 years old and had 16.6 years of education, and were majority female (54.1%) and White (87.7%). A total of 256 falls were recorded in the 12 months following the Year 1 home visit, with a median of 1 fall (Table 2). All participants were cognitively normal at baseline (CDR = 0).

**Table 2:** Participant characteristics at Year 1

	*n* = 205
Age, *M* ± SD	74.8 ± 5.8
Gender, female, n (%)	111 (54.1)
Race, n (%)	
Black	24 (11.7)
White	180 (87.8)
Two or more races	1 (0.5)
Years of education, *M* ± SD	16.6 ± 2.4
Falls	
Total, n	256
In 12 months, median [IQR]	1 [0–2]
Balance and strength, median [IQR]	
CST number of stands	12 [10–14]
POMA total score	26 [24–27]
Vision, median	
Near visual acuity	20/25
Low contrast visual acuity	20/16
Fear of falling, median [IQR]	
FES-I total score	8 [7–10]
Home hazards, n (%)	
≥4 home hazards	5 [3–8]
Sensation, n (%)	
Vibration sensation impaired	119 (58)
Sharp sensation impaired	98 (47.8)
Medication, n (%)	
≥4 prescription medications	97 (47.3)

Note. IQR = interquartile range; CST *=* Chair Stand Test; POMA = Tinetti Performance Oriented Mobility Assessment; FES-I = Short Falls Efficacy Scale-International

A frequency analysis of recommendation type and follow through was assessed for individuals who fell versus those who did not (Table 3). Very few participants (*n* = 16; 8%) did not have any fall risks in the 6 domains and did not receive any recommendations. The 127 older adults who fell received 359 recommendations, while the 78 older adults who did not fall received 179 recommendations. Individuals who fell were significantly more likely to receive recommendations to discuss fall prevention classes with their doctor (fear of falling; *p* = 0.01) and have a doctor or pharmacist review their medications (medication; *p* = 0.004) than those who did not fall. They were also more likely to receive recommendations to remove hazards in their home (home hazards) and have their lower extremity sensation tested (sensation), though these between-group differences were not statistically significant. There were no differences in follow through with recommendations between those who did and did not fall.

**Table 3:** Types of recommendations and follow through with recommendations for fallers and non-fallers

	Fell (*n* = 127) n (%)	Did not fall (*n* = 78) n (%)	Proportion difference [95% CI]	*p*-value
Total number of recommendations	359	179	—	—
Total follow through with recommendations	220 (61.3)	100 (55.9)	0.05 [-0.03, 0.14]	0.23
No recommendations	11 (8.7)	5 (6.4)	0.02 [-0.05, 0.1]	0.56
Balance recommendation	54 (42.5)	27 (34.6)	0.08 [-0.05, 0.21]	0.26
Balance follow through ^a^	31 (58.4)	15 (57.7)	0.008 [-0.22, 0.24]	0.95
Vision recommendation	29 (22.8)	18 (23.1)	0.002 [-0.12, 0.12]	0.97
Vision follow through ^a^	25 (86.2)	12 (75.0)	0.11 [-0.13, 0.36]	0.35
Fear of falling recommendation	31 (24.4)	8 (10.3)	0.14 [0.04, 0.24]	0.01*
Fear of falling follow through ^a^	7 (23.3)	2 (25.0)	− 0.02 [-0.35, 0.32]	0.92
Home hazards recommendation	83 (65.4)	44 (56.4)	0.09 [-0.05, 0.23]	0.20
Home hazards follow through ^a^	46 (56.8)	17 (41.5)	0.15 [-0.03, 0.34]	0.11
Sensation recommendation	92 (72.4)	55 (70.5)	0.02 [-0.11, 0.15]	0.77
Sensation follow through ^a^	55 (61.8)	32 (60.4)	0.01 [-0.15, 0.18]	0.87
Medication recommendation	70 (55.1)	27 (34.6)	0.21 [0.07, 0.34]	0.004**
Medication follow through ^a^	56 (82.4)	22 (91.7)	− 0.09 [-0.24, 0.05]	0.27

^a^*n* = 203

*p < 0.05, **p < 0.01

Overall, participants reported high levels of satisfaction with receiving their fall risk results via the HRC (93%; *n* = 186). The majority (90%; *n* = 180) found receiving their fall risk results to be beneficial. Few participants (20%; *n* = 40) shared their fall risk results with their doctor as recommended (see Supplementary Table 2).

## Discussion

This study examined the impact of providing tailored recommendations based on results from an OT fall risk screening, follow through with those recommendations to reduce the risk of falling, and satisfaction with receiving personalized information about fall risk in community-dwelling older adults. Overall, most participants received fall risk recommendations to reduce home hazards, check their lower extremity sensation, or have a medication review; they were most likely to follow through with getting an annual eye exam or reviewing medications with a health care provider. Participants who fell were significantly more likely to receive recommendations for discussing fall prevention programs with their doctor and reviewing their medications than those who did not fall. Those who fell were also more likely to receive recommendations to remove hazards in their home, though the difference was not significant. Of the recommendations provided, participants were most likely to follow through with recommendations for medication (82%), home hazards (57%), and fear of falling (23%). Overall, older adults who fell followed through with 61% of recommendations compared to 55% for those who did not fall. These results suggest that providing fall risk results and tailored recommendations alone is not sufficient to encourage follow through with recommendations, effect change for engaging in fall prevention behaviors, or reduce fall risks in 1 year. Future fall prevention efforts for older adults who have a history of falls should address home modification and hazard removal delivered by an occupational therapist [7], implementation of fall prevention classes, medication review, and continued encouragement to ensure follow through with fall prevention strategies, including notifying their primary care physician directly about any falls [13].

The majority of participants were satisfied with receiving their fall risk results in the HRC, demonstrating acceptability of using the HRC to convey fall risk information and tailored recommendations among community-dwelling older adults. However, only 20% of participants reported sharing their fall risk results with their doctor. It is unclear why the participants did not share the results. It is possible that their doctors are already aware of the risks, or participants may believe that falls are a normal part of aging or that they are not personally at risk for falls [8, 9]. Over 60% of participants in this study experienced one or more falls, which is greater than the approximately 30% of older adults who report falling each year based on annual national surveying [1]. It is possible that this higher prevalence of falls may be attributed to greater accuracy and enhanced recall through monthly fall monitoring compared to annual fall monitoring [23, 24]. Providing additional opportunities for older adults to report falls may result in increased awareness of their own fall risks and allow for provision of fall prevention education, programs, and studies to decrease falls and increase participation among community-dwelling older adults.

This study has several limitations that impact interpretation of the results. First, the majority of participants were female and White, which limits the generalizability of these findings. Additionally, this study did not ascertain existing behaviors related to recommendations provided; participants already may have been aware of their fall risks related to these 6 domains or engaging in regular fall prevention behaviors, such as getting annual eye exams or reviewing medications with health care providers, prior to this study. Therefore, follow through with recommendations cannot be ascribed solely to the HRC. Additional information is needed to compare existing versus post-HRC follow through with fall risk recommendations. While most participants found receiving their fall risk information from the HRC to be beneficial, it is possible that the low rate of sharing HRCs with primary care physicians could be due to the length of time between receiving the HRC and follow-up with a study team member 1 year later. To address this concern, future studies could investigate the impact of more frequent contact with participants or providing reminders to follow through with HRC recommendations. Additional opportunities to report follow through, as well as information about the reasons for not following through with the recommendations, may provide valuable information for addressing fall risks and enhancing follow through with recommendations in the future. Last, due to the COVID-19 pandemic, some participants may have been limited in their ability to follow through with the recommendations or may not have remembered whether they followed through with the recommendations because of the extended time between Year 1 and follow-up.

Future studies should utilize rigorous designs, such as randomized controlled trials, to test the efficacy of the HRC as a potential intervention for reducing falls and promoting utilization of fall prevention behaviors. Additionally, inquiries should ascertain older adults’ reasons for lack of follow through with fall risk recommendations in the HRC as well as provide reminders for follow through between visits. More frequent follow-up regarding fall risk recommendations may impact one’s decision to take action for engaging in fall prevention behaviors. It may also be beneficial to quantify any interactions with medical providers regarding fall risks, as this may influence participants’ decisions to follow through with recommendations. Future studies utilizing the HRC should examine the relationship between acting on recommendations and the time to experiencing a fall to further examine the role of the HRC recommendations and follow through in reducing risk of falls. Finally, future work should investigate supplemental information to the HRC that may improve its effectiveness in reducing falls risk, such as referrals for fall prevention treatment with an OT provider.

## Conclusions

These findings highlight the importance of addressing fall prevention strategies for at-risk older adults. Older adults may benefit from additional support and encouragement when receiving fall risk recommendations, especially reducing home hazards, providing fall prevention classes, and promoting exercises to improve balance and gait. Additional research is needed to examine follow through and awareness of fall risks among community-dwelling older adults.

### Electronic supplementary material

Below is the link to the electronic supplementary material.

**Supplementary Material 1: Supplementary Table 1.** Chi-square test of recommendations between fallers and non-fallers. **Supplementary Table 2.** Satisfaction with receiving fall risk results

## Data Availability

The datasets analyzed during the current study are available from the corresponding author on reasonable request.

## Abbreviations

ADRC: Alzheimer Disease Research Center
CDR: Clinical Dementia Rating
HRC: Health report card
OT: Occupational therapy
