# Supplementary material for: Examining the impact of a health report card on follow through with fall risk recommendations: an observational study
Source: BMC Geriatr. 2024 Feb 16;24:166. doi: 10.1186/s12877-024-04686-y (PMC10873987; doi:10.1186/s12877-024-04686-y)
Supplement: Supplementary file 1 — Supplementary Material 1: Supplementary Table 1. Chi-square test of recommendations between fallers and non-fallers. Supplementary Table 2. Satisfaction with receiving fall risk results [file 12877_2024_4686_MOESM1_ESM.docx]

**Supplementary Tables**

**Supplementary Table 1.** Chi-square test of recommendations between fallers and non-fallers

| Recommendation | Chi-square | p-value |
| --- | --- | --- |
| Total recommendation follow through | 1.4534 | 0.228 |
| No recommendations | 0.36982 | 0.5431 |
| Balance | 1.4207 | 0.2333 |
| Balance follow through | 0.0045697 | 0.9461 |
| Vision | 6.8688^e-05^ | 0.9934 |
| Vision follow through | 0.88595 | 0.3466 |
| Fear of falling | 6.4736 | 0.01095 |
| Fear of falling follow through | 0.0097063 | 0.9215 |
| Home hazards | 1.9216 | 0.1657 |
| Hazards follow through | 2.5606 | 0.1096 |
| Sensation | 0.1896 | 0.6632 |
| Sensation follow through | 0.028242 | 0.8665 |
| Medications | 8.5722 | 0.003413 |
| Medications follow through | 1.1927 | 0.2748 |

|  | Frequency (%) n=201 |
| --- | --- |
| Satisfied with receiving the health report card | 186 (92.5) |
| Found health report card beneficial | 180 (89.6) |
| Shared health report card with their doctor | 40 (19.9) |

**Supplementary Table 2.** Satisfaction with receiving fall risk results
